# The Impact of Community-Based Health Insurance on Household's Welfare in Chilga District, Amhara Regional State, Ethiopia

**DOI:** 10.3389/fpubh.2022.868274

**Published:** 2022-06-02

**Authors:** Dagmawe Menelek Asfaw, Sirage Mohammed Shifaw, Atinkugn Assefa Belete, Setognal Birara Aychiluhm

**Affiliations:** ^1^Department of Economics, College of Business and Economics, Samara University, Samara, Ethiopia; ^2^Department of Public Health, College of Medicine and Health Science, Samara University, Samara, Ethiopia

**Keywords:** CBHI, welfare, probit, PSM, Chilga, Ethiopia

## Abstract

Household welfare is depleted by catastrophic health expenditure by forcing families to reduce the consumption of necessary goods and services, underutilization of health services, and of finally falling into the poverty trap. To mitigate such problem, the Government of Ethiopia launched CBHI schemes. Therefore, this study investigates the household welfare impact of Community based health insurance (CBHI) in the Chilga district. A multi-stage sampling technique was used to select 531 households (of which 356 were treated and 175 control groups). Probit and propensity score matching (PSM) were used to analyze the data. Probit model revealed the following: Level of education, access to credit, chronic disease, insurance premium, awareness, distance to health service, and health service waiting time are significant determinates for being insured in CBHI. The PSM method revealed that the insured households associated with visits increased by 2.6 times, reduced per-capita health expenditure by 17–14% points, increased the per-capita consumption of non-food items by 12–14% points, increased the per-capita consumption of food items by 12–13% points in a given matching algorithm compared to the counterparts. Therefore, CBHI has enhanced service utilization by reducing per-capita health expenditure and increasing consumption per-capita, in general, it improved household welfare. To this end, the results of this study suggested that the government (ministry of health) and concerned bodies (such as NGOs) should extend the coverage and accessibility of CBHI schemes, create aware to the society about CBHI, and subsidize premium costs of the poor.

## Introduction

Globally, about 100 million people were projected to be living under the poverty line and hence they were likely to face poverty due to health catastrophic spending (impoverishing health expenditures) (1). Catastrophic health expenditures (CHE) have a greater impact on household welfare around the globe, which is forcing families to reduce the consumption of necessary goods, underutilization of health services, and finally fall into the poverty trap and distract their social wellbeing (2).

In underdeveloped countries, catastrophic health expenditures hurt households' income, impairment households' welfare, push them into penury, aggravate their poverty, and obstruct health service utilization (3). Finally, the amalgamation impact of such effects would be a big macroeconomics problem for the nation. Globally, about 800 million people expose CHE in 2010 (1), and also CHE estimates in 133 countries found that the incidence had increased in almost half of these countries over the last decade (4).

Different studies also support this; for example, the study by Wei et al. (2) found that a percentage increment in the per-capita medical expenses of a household, the per-capita food consumption expenses of a household diminished by 7.6%, and also Leive and Xu (5) studied on 15 African countries, including Ethiopia, revealed that there was tradeoff relationship between health expenditure and consumption expenditure. Similarly, Shikuro et al. (6) and Kiros et al. (7) found that the catastrophic health expenditures on households were 21.5 and 22.5%, respectively, and such expenditures also devastate the consumption expenditures and social wellbeing.

Health care expenses in Ethiopia also hurt and have a long-term impact on the economic status of the majority of poor households. In Ethiopia, out-of-pocket health payments constituted about 33% of total health spending in Ethiopia, which was higher than that of other African and OECD countries which account for 30.6 and 19.6% of the total health expenditure (8), and such payments were particularly difficult for 24% of the people that lives under extreme poverty and also about 18% who needed health service were not able to access because of the financial constraint and expensiveness of health service (9).

To mitigate such nation-wise problems, the Ethiopian government launched a pilot voluntary community-based health insurance (CBHI) scheme in 2011 in 13 districts, aimed to protect low-income society from the impoverishing effects of catastrophic health expenditures, improve the demand for health service utilization, and widen the income source from domestic sources for the health care sector (10). The registration fee per household was Birr 3; the premium also allotted as Birr 132 per household per year plus an addition Birr 30 per person/year for the dependents more than 18 years of age; and also 70% of the targeted subsidy from the region and 30% from the district (10). The scheme covers almost every district of Amhara Regional State, including the Chilga district. According to of Chiliga district community-based health insurance branch office in 2022, a total of 4,721 households were insured in this scheme, in line with this insurance premium for the households per annum whose family size is between 1–5, 6–7, and 8 and more is going to be 400, 500, and Birr 530, respectively.

The previous empirical works have investigated the fundamental factor that determined CBHI enrolment and impacts on the health care utilization in Ethiopia (3, 8, 11–14), and so on; however, there is very little empirical literature that has been conducted on the welfare impact of CBHI in Ethiopia (15, 16), and also no study has been conducted so far in this study area about CBHI impact on household welfare. In addition, most studies used per-capita income or expenditure to measure the welfare impact of CBHI rather than non-monetary social goods, such as health service utilization. Measuring the welfare only in terms of monetary aspect does not show the true impact of CBHI on welfare; therefore, it should be considered non-monetary aspects, such as a social good (service utilization). Similarly, measuring welfare in terms of income is difficult especially in the developing countries because the household source of income is too diversified and seasonally volatile; and it is under-reported (17). Therefore, this study analyzes the impact of CBHI on household welfare by considering monetary aspects as well as social goods of welfare measurement in Chilga district, Amhara Regional State, Ethiopia.

## Materials and Methods

### Study Design and Description of the Study Area

A community-based cross-sectional study was conducted in Chilga district, Amhara Regional State, Central Gondar Zone, Ethiopia. This was because, this district is a highly-populated area and economic and social interaction center for different districts. Chilga is a district of the new Central Gondar Zone and a stopping point on the famous Gondar–Sudan trade route and is found 61 km west of Gondar town on the way to Metemma. Chilga district shares the border with Takusa district in the south, Metemma district in the west, Tach Armachiho district in the north, Lay Armachiho in the northeast, and Dembia district in the east. There are three main towns in this district, namely, Aykel, Seraba, and Wohni. The district's elevation ranges between 1,000 and 1,500 m above sea level. The agroecology of the district is Kola and Woinadega, which constitute around 67 and 33%, respectively (18).

The district gets a minimum of 995-mm and a maximum of 1,175-mm annual rainfall and 27°C mean daily average temperature. Land in this district shows that 22.3% forest or shrubland, 21.7% is arable, 1.9% pasture, and the remaining 54.1% is considered degraded or other. In the district, 221,462 people live, and among them 112,054 are men and the remaining 109,408 are women. Also, 20,745 or 9.37% are urban inhabitants while 90.63% are rural inhabitants (18). A total of 47,336 households were found in this district; among these, there were 4.68 persons lived per household on average and there were 45,352 housing units. The majority of the inhabitants follow Ethiopian Orthodox Christianity, which constitutes 96.7%, while 3.1% of the population practices Islamic religion (19).

### Sampling Technique and Sample Size

*A multistage sampling technique*. In the first stage, from the total 16 kebelles^1^ in the Chilga district insured in the CBHI program, 4 kebelles, namely, *Chandiba–Debega, Kuwak–Gebeluha, Chalia–Deber, and Dangura* were selected as purposive sampling based on the total number of households insured in the CBHI scheme. In the second stage, a total of 531 sampled households were allotted to each selected kebelles based on their population proportion. The total amount of treated and control samples from each sampled kebelles was determined by using the total percentage share of insured and uninsured households in the Chilga district CBHI scheme (from the total households of 3,566 or 33% households insured and 7,239 or 67% households uninsured in CBHI program at Chilga district). In the third stage, select 175 treated and 316 control samples from each sampled kebelles by using systematic random sampling (see Table 1).

**Table 1:** Sampling techniques and sample size.

**Name of sampled kebelles**	**Total number of households**	**Sampled households**		
		**Total**	**Treated**	**Control**
Chandiba–Debega	1,847	184	61	123
Kuwak–Gebeluha	1,236	123	41	82
Chalia–Deber	1,232	122	40	82
Dangura	1,026	102	34	68
**Total**	**5,341**	**531**	**175**	**356**

*Source: Chilga district CBHI branch office and own computation, 2022*.

The intended sample sizes were determined by using Kothari (20) sample size determination formula as follows (Equation 1).


(1) $$\begin{array}{r} n=\frac{Z^{2}pq}{e^{2}}=\frac{{\left(1.96\right)}^{2}\left(0.67\right)\left(0.33\right)}{{\left(0.04\right)}^{2}}=530.86\approx 531 \end{array}$$

where *n* is the sample size; *z* = 1.96 to achieve 95% the level of confidence; according to the report of Chilga district CBHI branch office in 2022, the share of the total insured household in CBHI at Chilga district was 67%; therefore, *p* = 0.67; *q* = 0.33; *n* = 531 is the sample size; *e* is the tolerant marginal error defined as 0.04, that is, 4% maximum discrepancy results between the sample and the general population (21).

### Sources and Methods of Data Collection

Primary and secondary data sources were employed. Primary data, which was collected from 531 sampled households from four sampled kebelles by a structured and semi-structured questionnaire that addressed demographic, socioeconomic, institutional, and health-related characteristics of the sampled households through a team of four trained enumerators of health extension workers for each sampled kebelles. The primary data was also collected from observation and key informant interviews with CBHI district coordinators, religious leaders, kebelle representatives, health extension workers, kebelle cabins, and other concerned bodies. The secondary data were collected from published and unpublished documents (CSA, journals, ministry of health, health bureau, and official reports).

### Analytical Framework

Analyze the data collected from 531 sample respondents by using two statistical methods. First, descriptive statistical methods, such as arithmetic means, standard deviations, percentages, and frequency, were used to describe and assess the socioeconomic characteristics, institutional, market characteristics of sampled households in the study area; and inferential statistics method that was independent *t*-test for continuous variables and the Chi-squared test for a categorical variable were applied for the analysis to describe the statistically significant differences between the treated and the control with regard to covariates.

The second analysis was done using the econometric analysis approach to examine the impact of CBHI on household welfare in the study area. Khandker et al. (22) impact evaluations are examines and measure actual impacts of the program/project after intervention on beneficiary societies (*ex post*). And also it can take place before the program/project intervention in order to predict likelihood impact of such program intervention on the host societies given covariates (*ex ante*). *Ex ante* and *ex post* impacts of a program can be addressed using a variety of quantitative approaches, but there are two main types: randomized experimental designs and quasi-experimental designs (non randomized).

Randomization is a method in which the selection of the treatment and the control groups is random within some well-defined set of people. Experimental designs work on random nature samples and also randomly allocate the intervention among treated and control groups, which are statistically equivalent to one another, when provided with appropriate sample sizes. Quasi-experimental (non-random) methods can be used to evaluate by construct treatment and counterfactual comparison groups. One of the quasi-experimental methods of data analysis is propensity score matching (PSM). Matching is a statistical technique that are attempting to find a non-treatment comparison group for every possible unit under the treatment unit that has the most similar characteristics possible. However, matching is difficult as it increases the number of characteristics and dimensions against which one wants to match units or it is called the curse of dimensionality (23).

Fortuitously, the problem of the curse of dimensionality could be easily resolved by using a method of propensity score matching (24). Propensity score matching method is a statistical matching technique that attempts to estimate the effect of policy intervention on outcome variables by accounting for baseline observed characteristics (covariates) and the probability of participating in the intervention (propensity score). Propensity score matching is a method that matched the treatment group with the control group based on the closest propensity score; these closest units become the comparison group and are used to produce an estimate of the counterfactual (25). The PSM tries to mimic the randomized assignment to treatment and comparison groups by choosing for the comparison group those units that have similar propensities to the units in the treatment group (26).

The application of the PSM method can be conducted by the following steps: The first step is to run a probit or logit model for the participation equation, then predict the probability of participation in the intervention (it is called propensity score). Second step is defining the region of common support (treatment observations have comparison observations “nearby” in the propensity score distribution), conditional independence (states that a given set of observable covariates that are not affected by treatment), potential outcomes are independent of treatment assignment, and balancing tests (the treatment and control groups must be balanced in that similar propensity scores are based on similar observed characteristics). Third step is matching participants to non-participants by using different matching techniques, such as nearest-neighbor matching, radius matching, stratification or interval matching, Kernel matching, and others. Fourth step is checking the quality of matching by using different methods, such as mean bias, *t*-test, pseudo *R*^2^, likelihood test, and joint/overall significant test (24); finally estimating the effect of treatment on treated group.

### Model Specification

Suppose *T*_*i*_ is treatment (be equal to 1 if sampled households have insured in CBHI and 0 if not insured), *x*_*i*_ is baseline characteristic of sample households, *Y*_*i*_(1) is the outcome variable for the *i*th households who have CBHI and *Y*_*i*_(0) is the outcome variable for *i*th households who do not have CBHI, Δ*Y* represents the impact of CBHI on a sampled household welfare (treatment effect), which is stated in Equation (2) as follows:


(2) $$\begin{array}{r} \text{Δ}Y=Y_{i}\left(1\right)/x_{i}-Y_{i}\left(0\right)/x_{i} \end{array}$$

The average treatment effect (ATE) of CBHI is also represented in Equation (3) as follows:


(3) $$\begin{array}{l} ATE=E\left[Y_{i}\left(1\right)-Y_{i}\left(0\right)/x_{i},T_{i}\right]=\;E[Y_{i}\left(1\right)/x_{i},T_{i}=1]\\ \;-E[Y_{i}\left(0\right)/x_{i},T_{i}=0] \end{array}$$

However, such a comparison might not capture the true impact of the CBHI on household welfare, because the baseline characteristics of the treated and control groups are statistically different. If we have to use a single household for both the treated and control groups simultaneously, we are not proceeding with the analysis because households can only be in one group at a time and only one of the potential outcomes can be observed at a time (27). The solution is to construct the counterfactual for the treated households, which means calculating the outcome of treated observation if they had not been treated (26). Therefore, the average treatment effect on the treated (ATT) is depicted in Equation (4) as follows:


(4) $$\begin{array}{l} ATT=E\left[Y_{i}\left(1\right)-Y_{i}\left(0\right)/x_{i},T_{i}\right]=\;E[Y_{i}\left(1\right)/x_{i},T_{i}=1]\\ \;-E[Y_{i}\left(0\right)/x_{i},T_{i}=1] \end{array}$$

*ATT* is the difference between expected welfare impact with and without CBHI for those who participated in CBHI (28).

To operationalize PSM, we follow two steps as follows: First, model the participation decision of CBHI utilizing probit models as a choice-dependent variable to estimate propensity score as depicted in Equation (5) as follows:


(5) $$\begin{array}{l} T_{i}=\beta_{0}+\beta_{1}age_{i}+\beta_{2}gen_{i}+\beta_{3}hhsize_{i}+\beta_{4}edu_{i}+\beta_{5}dsthlt_{i}\\ \;+\beta_{6}credit_{i}+\beta_{7}mrtst_{i}+\beta_{8}chrodis_{i}+\beta_{9}insuprm_{i}\\ \;+\beta_{10}awarns+\beta_{11}timesrv_{i}+\beta_{12}child18_{i}\\ \;+\beta_{13}adult64_{i}+\varepsilon_{i} \end{array}$$

(6) $$\begin{array}{rcl} T_{i} & = & \beta_{0}+X_{i}+\varepsilon_{i} \end{array}$$

After the matching was successful and passed all the required and necessary steps, we can estimate the ATT as depicted in Equation (7) as follows:


(7) $$\begin{array}{r} HHwelfare_{i}=\alpha_{0}+\alpha_{1}T_{i}+Pscore_{i}+X_{i}+u_{i} \end{array}$$

The definitions of dependent and independent variables used in the PSM model to analyze the impact of CBHI on household welfare are presented for the *i*th sampled households in Table 2.

**Table 2:** Definition of dependent and explanatory variables.

**Variables**	**Definition**	**Measurement**
*X*_*i*_	Explanatory variables	
*Pscore*_*i*_	Estimated propensity score	
*T*	Treatment (CBHI)	1 = insured in CBHI, 0 = uninsured
*age*	Household head age	Year
*gen*	Sex of household head	0 = Female, 1 = Male
*hhsize*	Household size	Headcount
*edu*	Educational level of households	0 = not able to read and write, 1 = able to read and write, 2 = primary school (1–8), 3 = secondary school and above
*dsthlt*	Distance to nearest health services	Kilometer
*Credit*	Access to credit	0 = no access of credit, 1 = access of credit
*mrtst*	Marital status	0 = single, 1 = married, 2 = divorced, and 3 = windowed
*chrodis*	Chronic disease in the household	0 = absent, 1 = present
*insuprm*	Insurance premium	0 = unaffordable, 1 = somewhat affordable, 2 = easily affordable
*awarns*	Awareness about CBHI	0 = good, 1 = bad
*timesrv*	Health service waiting time	Hour
*child 18*	Present of children <18 age	Headcount
*adult 64*	Present of adult more than 64 age	Headcount
*HHwelfare*	Household welfare	Health service utilization (outpatient and inpatient visit)
		ln per-capita expenditure of health
		ln per-capita consumption of non-food and beverage item
		ln per-capita consumption of food exclude beverage item

### Ethical Consideration and Consent to Participate

Ethical clearance was obtained from the College of Business and Economics, Samara University. Confidentiality of the information was secured by excluding respondents' identifiers, such as names, from the data collection format. Finally, verbal informed consent was obtained from those who were in the Chilga district and willing to participate in the study. Moreover, the results were recommended to be disseminated by the responsible bodies who were involved in health sectors.

## Results and Discussion

### Descriptive Analysis

The descriptive analysis of this study was conducted by descriptive statistics (means, standard deviation, frequencies, and percentages) and inferential statistics (independent *t*-test and Chi-squared test) to assess, compare, and check the relationship of the dependent variables across the independent variables. The descriptive comparison of the categorical variables over dependent variables based on frequency counts and the Chi-squared test is presented in Table 3.

**Table 3:** Baseline characteristics of a sampled household before intervention.

**Variables**	**Households' status CBHI**		**Total (*n* = 531)**	***t*-test/ x^2^**
	**Uninsured *n* = 356 (67%)**	**Insured *n* = 175 (33%)**		
Sex of household head (Male)^a^	310 (87.0%)	165 (94.0%)	488 (92.0%)	5.74
Educational level of household head (able to read and write)^a^	142 (39.8%)	76 (44.0%)	218 (41.0%)	19.94^***^
Educational level of household head [primary school (1–8)]^a^	112 (31.0%)	56 (32.0%)	168 (31.6)	
The educational level of household head (secondary school and above)^a^	30 (8.5%)	16 (9.1%)	46 (8.6%)	
Credit (Access of credit)^a^	110 (31.0%)	58 (33.1%)	168 (31.6%)	12.36^**^
Marital Status (Married)^a^	288 (81.0%)	151 (86.0%)	439 (82.6%)	1.63^***^
Marital Status (Divorced)^a^	31 (8.7%)	14 (8.0%)	45 (8.40%)	
Marital Status (Windowed)^a^	28 (7.8%)	7 (4.0%)	35 (6.6%)	
Chronic disease in the household (Present)^a^	117 (33.0%)	65 (37.0%)	183 (34.2%)	2.52
Insurance premium (Somewhat affordable)^a^	85 (23.9%)	57 (32.5%)	142 (26.7%)	9.56^**^
Insurance premium (easily affordable)^a^	202 (57.0%)	102 (58.2%)	304 (57.2%)	
Awareness about CBHI^a^	281 (79.0%)	144 (82.2%)	425 (80%)	1.56
The presence of child <18 years^a^	210 (59.0%)	108 (61.7%)	318 (59.8%)	7.01^**^
The presence of young higher than 64 years^a^	35 (10.0%)	23 (13.0%)	58 (10.9%)	3.24
Age of household head^b^	42 (0.86)	44 (0.64)	42.9 (0.74)	4.88^***^
Household size^b^	6.2 (0.21)	7 (0.12)	6.5 (0.18)	1.77
Distance to nearest health service^b^	12 (0.19)	10 (0.29)	11 (0.23)	6.55^***^
Health service waiting time^b^	2.3 (0.74)	1.2 (0.10)	1.6 (0.14)	3.66^***^
Household food expenditure^b^	6,363 (62.07)	7,872 (67.25)	6,860 (236)	5.69^**^
Household non-food expenditure^b^	4,039 (47.65)	6,008 (56.32)	4,688 (352)	9.63^***^
Household health expenditure^b^	3,012 (30.29)	2,628 (40.28)	2,885 (587)	8.52^***^

*^“a”^ expressed in number (frequency) and ^“b”^ expressed mean (SD)*.

^***^ and ^**^ denote 1 and 5 levels of significance.

*Source: Own computation, 2022*.

The share of male-headed households in insured and uninsured groups of sampled households are 94 and 87%, respectively. Regarding the educational background of insured sampled households, 44% can read and write, 32% achieved primary education, 9.1% attained secondary education and above, and the remaining 15% are not able to read and write. Therefore, education brings know-how and awareness for CBHI (see Table 3). The Chi-squared test suggests a positive association between household education and insured in CBHI. In comparison, Table 3 reveals that about 31.6% of respondents had access to credit, 57 and 26% of sampled households say that the insurance premium is easily and somewhat affordable, respectively. This means that the households had access to credit and the insurance premium is also affordable; the households are eager to be insured in CBHI.

In the insured sampled households, 86% are married and in uninsured households, 81% of them are also married. This will be indicated that married households could have more families with health expenses and enthusiastic to participate in CBHI. The results under Table 3 reveal that 34.2% of the total sampled households had chronic diseases, among this 37% of the insured subsample and 33% of the uninsured subsample households had chronic diseases. Therefore, the higher the presence of chronic disease, the higher chance to be insured in CBHI. Also, 26% of the sampled households had awareness about CBHI with a higher percentage for insured groups (82.2%) relative to the uninsured groups (79%).

A child whose age is <18 years and an adult whose age is higher than 64 years exist in uninsured households, and these two categories are relatively higher (61 and 13%, respectively) than their counterparts (59 and 10%, respectively). The average age of households was 43, which indicated that they are within the economic working age. The average age for the insured and uninsured households was about 44 and 42 years, respectively. The average household size in the study was 7 people per household approximately, whereas the mean health service waiting time for insured and uninsured sampled households was 1.2 and 2.3 h, respectively. Availability of health services, such as health care center and clinic, hospital has contributed for insured in CBHI, that is for insured household health service is 10 km away from their dwelling, whereas, for the uninsured house, households health service avail is beyond 12 km from their home. On average, the household expenditure for health, food, and non-food items were Birr 2,885, 6,860, and 4,688 per year, respectively (see Table 3).

### Econometrics Analysis

#### The Determinants to Be Insured in CBHI

Before analyzing the econometrics model, the cross-check multi-collinearity test for both continuous and categorical with the help of variance inflation factor (VIF) and contingency coefficients (CC) for continuous and dummy variables, respectively, were conducted (29); the heteroskedasticity test was conducted using the Brushi–Pagan test (30); the omitted variable test was conducted using the Ramsey test; and the normality test was conducted using a Kernel density plot (31). In such a way, the test results justified that there was no strong collinearity between explanatory variables; the variance of error term was constant conditional on the chosen value of the explanatory variables; no omitted variables in the model; and the error term is normally distributed with its mean and variance.

In Table 4, probit regression is shown that education is a statistically and positively significant determinant for insured in CBHI. The marginal effect demonstrated that a household can read and write, accomplished primary school, and secondary school is about 13, 23, and 31%; and 3.2% more likely to get insured in CBHI than their counterparts (who cannot read and write). A household with awareness about CBHI is a 13% more probability to be insured in CBHI than its counterparts and the other things remain constant. This might be due to the fact that education brings knowledge and techniques for searching and understanding information and developing awareness. In line with this, households had an awareness about insurance principles and the functioning of the CBHI, they were more eager to be insured in CBHI packages. This finding is compatible with the previous studies (32–35).

**Table 4:** Probit model for the determinants to be insured in CBHI.

**Variables**	**Coefficient**	**Std. err**.	**Marginal effects**	**Std. err**.	***Z***
Sex of household head (Male)	−0.056	0.034	−0.020	0.011	1.767
Educational level of household head (able to read and write)	0.356	0.127	0.136^***^	0.048	2.834
Educational level of household head [primary school (1–8)]	0.687	0.345	0.234^**^	0.107	2.191
Educational level of household head (secondary school and above)	0.896	0.296	0.314^***^	0.090	3.497
Credit (Access of credit)	0.642	0.302	0.211^**^	0.099	2.126
Marital status (Married)	0.963	0.752	0.267	0.191	1.401
Marital status (divorced)	−0.903	0.505	−0.239	0.131	1.818
Marital status (Windowed)	−0.624	0.752	−0.187	0.206	0.910
Chronic disease in the household (present)	0.605	0.148	0.201^***^	0.049	4.138
Insurance premium (somewhat affordable)	0.236	0.076	0.082^***^	0.026	3.105
Insurance premium (easily affordable)	0.593	0.268	0.197^**^	0.089	2.223
Awareness about CBHI	0.362	0.178	0.134^**^	0.064	2.084
The presence of child <18 years	0.625	0.921	0.218	0.287	0.759
The presence of young higher than 64 years	0.603	0.341	0.169	0.095	1.788
Age of household head	0.025	0.019	0.008	0.006	1.316
Household size	0.069	0.150	0.022	0.027	0.840
Distance to nearest health service	−0.839	0.402	−0.275^**^	0.131	2.097
Health service waiting time	−0.487	0.159	−0.157^***^	0.051	3.093
Number of observations	531				
LR Chi^2^ (18)	125.58				
Prob > Chi^2^	0.000				
Pseudo *R*^2^	0.29				
Sensitivity	90.23				
Specificity	75.21				
Total correctly classified (%)	85.32				

^***^ and ^**^ denote 1 and 5 levels of significance.

*Source: Own computation, 2022*.

Access to credit was a positive and significant determinant of being insured in CBHI (see Table 4). This finding is similar with other studies (36, 37). Credit is used as a means of financial constraints for a poor household for affordable insurance premium, therefore, a household had access of credit makes them insured in CBHI compared to a household without access of credit. However, a previous study (38) found that the household with availability of credit package could enable them to think and give more attention to repay debt rather than to participated in the health insurance package; therefore, access of credit is inversely related to the demand to be insured CBHI scheme.

A household with poor health and chronic illnesses needs more frequent health service follow-up, which also brings an additional as well as unaffordable cost of health service. In such a way, such households were obliged to be insured in CBHI. The result under Table 4 revealed that those households who have a member with chronic disease have, 20% more probability to be insured in CBHI as compared to a household member without a member with chronic diseases, other things remain constant. Therefore, this result gives an insight that CBHI schemes in Ethiopia were prone to adverse selection. This finding is consistent with previous findings: Mirach et al. (34), Kwon (37), and Adebayo et al. (39).

In Ethiopia, most rural household were poor and more sensitive to the amount of insurance premium, in line with this unaffordable insurance premium is a means to exclude them from health insurance packages. Therefore, as Table 4 justified that when the insurance premium is easily and somewhat affordable, the likelihood of a household being insured is increased by 19 and 8%, respectively, compared to the base (unaffordable insurance premium); other things remain constant. This finding is supported by the result of the previous studies (40, 41).

A study (42) justified that a household far away from health care facilities and institutional rigidities health service system can play major roles in limiting insurance enrollment. The nearest distance to health service would increase the likelihood of participation and membership renewal and also decreases other indirect costs related to insurance schemes (37). As the health service is far away from home by a kilometer, the probability of households being insured is decreased by 27%; other things remain constant. This finding is in line with the previous studies (9, 13, 38, 40).

One of the measurements of better health utilization is timely delivered health service. As the diagnosis is on time and the service delivered time is shortened, the households will be interested to become a member in CBHI, unless they are not willing to be insured in CBHI packages. According to Ethiopian Health Insurance Agency (EHIA) in 2015, short waiting times are the major factor for households to be insured in CBHI. As Table 4 reports, the households are less likely to be insured when the waiting time for health services increased by a few hours. This finding is similar to the previous studies (43, 44).

#### The PSM Estimation for the Impact of CBHI on Household Welfare

**Figure 1:**
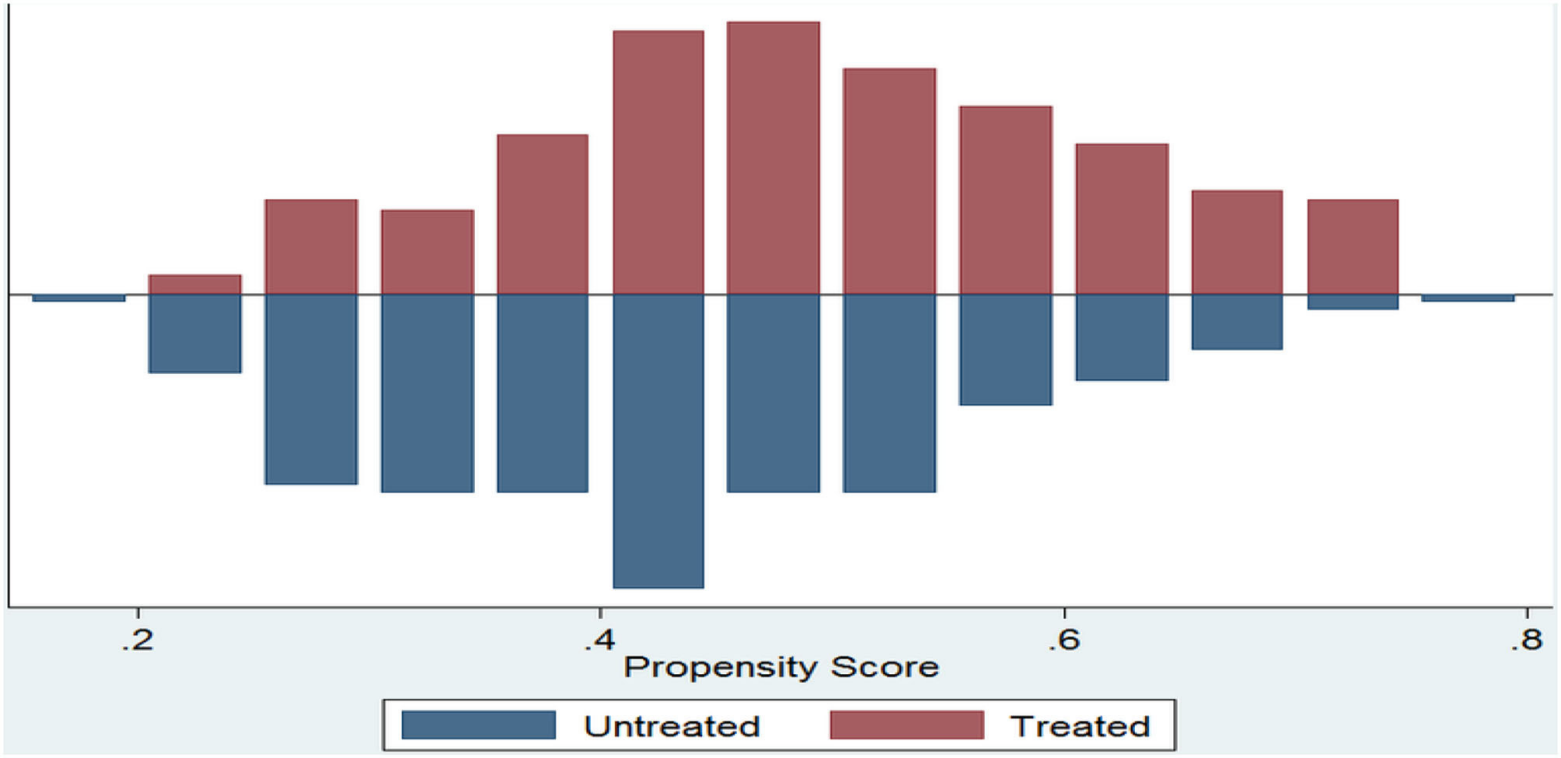
Common support of propensity scores. *Source:* Own computation, 2022.

The estimated propensity score by using the above probit model for the full sample varies from 0.005 to 0.804 with a mean value of about 0.41. A propensity score for insured households raged from the minimum of 0.205 to the maximum of 0.804 with a mean of 0.52 and for uninsured households ranged from the minimum of 0.005 to the maximum of 0.800 with a mean of 0.38. The region of common support is (0.205, 0.800). After estimating the propensity score and before matching, check the assumption of common support, overlapping assumption, and balancing properties to be achieved or not. Figure 1 indicated that the common support condition is achieved and there is substantial overlap in the distribution of the propensity scores of the insured (treated) and uninsured (control or untreated) groups.

After the above assumption is addressed, the next step is matching the insured (treated) group to the uninsured (control or untreated) group based on their propensity score with the help of different algorisms, such as radius, nearest-neighbor, and Kernel-based matching. However, after such matching, the quality of each matching should be tested using different techniques. In general, the quality of matching is to be measured by low pseudo *R*^2^-value, low LR Chi-squared value, low mean standardized biased (<5%), insignificant *p*-value, and low median biased (24). Table 5 reveal that Pseudo *R*^2^ decreased from 29.1% before matching to 0.1, 0.7, and 0.6% after nearest-neighbor, and Kernel-based and radius, respectively. Also, the LR Chi-squared and median biased significantly decreased after nearest-neighbor, and Kernel-based and radius. The mean standardized biased is decreasing and lower than 5% and *p*-value is insignificant in all matching algorism. Therefore, this indicates that PSM was a means of reducing selection bias due to observed characteristics.

**Table 5:** Overall matching quality indicators before and after matching.

**Matching algorisms**	**Sample**	**Pseudo *R*^2^**	**LR *x*^2^**	***p***	**Mean standardized bias**	**Median biased**
Baseline	Unmatched	0.291	125.58	0.000	24.8	23.7
Nearest neighbor	Matched	0.001	0.31	1.000	0.9	0.9
Kernel	Matched	0.007	3.17	1.000	3.1	2.8
Radius	Matched	0.006	3.15	1.000	3.6	3.5

*Source: own computation, 2022*.

In addition, a balance test was conducted by using the *t*-test and percentage biases. According to Table 6, the *t*-test indicated that before matching, most of the covariates are statistically significant and the percentage biased is >5%. This showed that the means difference of covariates between insured (treated) and uninsured (untreated) was statistically significant. However, after matching the *t*-test revealed that the mean differences are statistically insignificant and the percentage biased also <5%. Therefore, after matching, the baseline characteristics (covariates) between treated and control groups of sampled households have identical characteristics.

**Table 6:** Baseline characteristics of a sampled household before and after matching.

**Variables**	**Unmatched sample**				**Matched sample**			
	**Uninsured**	**Insured**	***t*-test**	**Bias (%)**	**Uninsured**	**Insured**	***t*-test**	**Bias (%)**
Sex of household head (Male)	0.870	0.940	2.56^**^	23.52	0.94	0.940	0.00	0.00
Educational level of household head (able to read and write)	0.391	0.440	2.06^**^	20.36	0.42	0.440	0.11	1.20
Educational level of household head [primary school (1–8)]	0.310	0.320	2.17^**^	21.35	0.32	0.320	0.00	0.00
Educational level of household head (secondary school and above)	0.085	0.091	1.23	13.26	0.085	0.091	0.19	1.80
Credit (Access of credit)	0.310	0.331	2.96^***^	25.36	0.325	0.331	0.11	1.20
Marital status (Married)	0.810	0.861	1.98^**^	19.23	0.861	0.861	0.00	0.00
Marital status (divorced)	0.087	0.008	4.36^***^	42.35	0.008	0.008	0.00	0.00
Marital status (Windowed)	0.078	0.004	1.09	11.32	0.034	0.004	0.28	2.30
Chronic disease in the household (present)	0.330	0.372	2.83^***^	24.23	0.365	0.372	0.11	1.20
Insurance premium (somewhat affordable)	0.239	0.325	3.04^***^	29.48	0.325	0.325	0.00	0.00
Insurance premium (easily affordable)	0.570	0.582	1.77	16.23	0.582	0.582	0.00	0.00
Awareness about CBHI	0.791	0.822	2.98^***^	28.53	0.822	0.822	0.00	0.00
The presence of child <18 years	0.590	0.617	1.63	15.25	0.617	0.617	0.00	0.00
The presence of young higher than 64 years	0.101	0.130	2.86	27.64	0.125	0.130	0.16	1.50
Age of household head	42.0	44.0	4.88^***^	43.2	44.28	44.37	0.08	0.80
Household size	6.2	7.0	1.77	16.23	7.06	7.09	0.09	1.10
Distance to nearest health service	12.0	10.0	6.55^***^	−42.12	10.04	10.07	0.07	0.70
Health service waiting time	2.3	1.2	3.66^***^	−38.32	1.16	1.20	0.25	2.60

^***^ and ^**^ denote 1 and 5 levels of significance.

*The matching algorithm was the nearest neighbor*.

*Source: Own computation, 2022*.

Table 7 illustrates the intensity of the impact of CBHI on welfare using three distinct PSM algorithms. CBHI had a positive and significant impact on health service utilization at a 1% level of significance. When a household is insured in CBHI packages, both outpatient and inpatient service utilization (visit) increase by 2.6 times compared to the uninsured. This might be due to the reason that CBHI mitigates financial barriers by reducing out-of-pocket money to access and utilize health services, in addition, CBHI packages are designed for households who are difficult to get access to the public, private, or employer health insurance packages and they live in the remote area, work in informal sectors and get subsistence income/wage. Therefore, CBHI improved the health service utilization (visit) of those households, this, in turn, contributes to enhancing the social wellbeing of households. This finding is similar to the other studies (3, 11, 45). Ethiopia's federal ministry of health conducted an evaluation report on the pilot CBHI scheme in 2015 and found that 72.3% of CBHI members visited health facilities while 69.3% of non-members also visit health facilities from the pilot study area. Another study (12) found that outpatient services utilization from public providers for CBHI members were 35% and for non-member also 22%. Similarly, s study (46) revealed that utilization of health services for CBHI members increased by 15% more than for CBHI non-members in Rwanda.

**Table 7:** The impact of CBHI on households' welfare.

**Outcome variables**	**Matching algorisms**					
	**NNM (Caliper** **=** **0.06)**		**KM (Bandwidth 0.06)**		**Radius (Caliper** **=** **0.06)**	
	**ATT**	**Std. err**.	**ATT**	**Std. err**.	**ATT**	**Std. err**.
Health service utilization (outpatient and inpatient visit)	2.62^***^	0.152	2.61^***^	0.151	2.6^***^	0.154
Ln per-capita health expenditure	−0.17^**^	0.013	−0.15^**^	0.015	−0.14^**^	0.032
Ln per-capita consumption of non-food and beverage items	0.14^***^	0.027	0.12^***^	0.026	0.11^***^	0.028
Ln per-capita consumption of food items	0.13^***^	0.022	0.12^***^	0.021	0.12^***^	0.022

*NNM, Nearest neighbor matching; KM, Kernel matching*.

^***^ and ^**^ represent that 1 and 5 levels of significance.

*Source: Own computation, 2022*.

Health care expenses are shocking and have an everlasting impact on the economic status of the majority of households in Ethiopia. In Ethiopia, out-of-pocket expenditure account for 38.5% of the total health expenditure, which was higher than that of other African and OECD countries which account for 30.6 and 19.6% of the total health expenditure, respectively (8). High medical expenditure cased not only reduced the ability to pay for necessities but also lead to a poverty vicious cycle. However, as a household insured in CBHI, their financial constrained are resolved, protected from catastrophe health expenditure, get access to health services at a relative minimum cost than uninsured households in the CBHI scheme, and this helps the households to escape from the poverty trap and improved welfare. According to Table 7, CBHI has a negative and significant effect on the household per-capita health expenditure at a 5% level of significance. The CBHI has decreased household per-capita health expenditure of the insured group by 17, 15, and 14 percentage points for nearest-neighbor, Kernel-based, and radius matching, respectively. This finding is similar to other studies (2, 14, 47, 48).

The results revealed that CBHI improves the welfare of households through consumption expenditure per capita. As listed in Table 7, CBHI had a positive and significant implication on per-capita expenditure on food, non-food, and beverage item at a 5% level of significance. Insured in CBHI schemes increases the per-capita consumption of non-food and beverages of households by 14, 12, and 11% points concerning each consecutive matching technique. Similarly, per-capita consumption of food items increased by 13, 12, and 12% points, respectively, of those three matching algorism for the households who are insured in the CBHI program. This might be because, as stated above CBHI is vital for reduced out-of-pocket health expenditure, mitigated a household from catastrophic health expenditure. This result increases the ability to purchase and consume for basic needs (such as food items) as well as non-food items. As households expand and achieved their psychological needs and also spend on non-food items their wellbeing is achieved. However, the impact of CBHI on per-capita expenditure on non-food is out weight the impact on food items. This may be due to the reason that most of the sample in this study were farmers; therefore, expenditure on non-food items was significantly higher than on food items. This result is supported by previous studies (8, 14, 49).

### Sensitivity Test

The finding under Table 7 assumes that the baseline characteristics of both treated and control groups are the same and observable or no unobserved characteristics have been existing. This is due to the reason that if there were unobserved confounders that have to be affected CBHI and welfare simultaneously, the intensity of ATT could be influenced by unobserved heterogeneity and yield biased results (14, 24). In line with this Rosenbaum, the sensitivity test checks the validity of this assumption. As indicated in Table 8, the empirical estimation of welfare indicators yields robust and insensitive to hidden bias up to at least five times the likelihood of being insured in CBHI.

**Table 8:** Rosenbaum sensitivity test.

**Gamma**	**Health expenditure/capita**		**Food expenditure/capita**		**Non-food expenditure/capita**		**Health utilization**	
	**Sig+**	**Sig–**	**Sig+**	**Sig–**	**Sig+**	**Sig–**	**Sig+**	**Sig–**
1	5.60E-11	0.000	7.80E-11	0.000	2.80E-14	0.000	2.30E-07	0.000
1.5	3.96E-10	0.000	9.30E-08	0.000	4.70E-12	0.000	9.80E-07	0.000
2	8.50E-07	0.000	5.80E-07	0.000	8.57E-08	0.000	1.89E-05	0.000
2.5	1.80E-05	0.000	4.70E-06	0.000	9.65E-07	0.000	5.60E-04	0.000
3	5.40E-04	0.000	8.30E-03	0.000	4.85E-05	0.000	1.25E-03	0.000
3.5	9.80E-04	0.000	1.40E-03	0.000	9.58E-05	0.000	6.90E-03	0.000
4	6.50E-03	0.000	1.85E-02	0.000	6.47E-04	0.000	9.80E-03	0.000
4.5	3.59E-01	0.000	3.26E-02	0.000	5.48E-03	0.000	1.87E-02	0.000
5	1.64E-02	0.000	6.05E-02	0.000	1.90E-02	0.000	3.25E-02	0.000
5.5	1.56E-01	0.000	1.46E-01	0.000	1.27E-01	0.000	2.37E-01	0.000
6	3.72E-01	0.000	2.62E-01	0.000	2.1E-01	0.000	5.67E-01	0.000

*Gamma—log odds of differential assignment due to unobserved factors*.

*Sig+: upper bound significance level, and Sig–: lower bound significance level*.

*Source: Own computation, 2022*.

For example, the gamma value of 5 produces an upper bound significance value of 0.0164, 0.019, and 0.0325 for health per-capita expenditure, non-food expenditure per-capita expenditure, and health utilization, respectively, this was below the standard threshold of 0.05. Likewise, for food expenditure per-capita expenditure, the gamma value of 5 offers an upper bound significant level of 0.06052, which was also significant at 0.1 thresholds. These results suggest that the results are insensitive to hidden bias that would increase the likelihood of being insured in CBHI by at least 5 folds.

## Conclusions and Recommendations

The objective of this study analyzed the impact of CBHI on household welfare. A probit model was employed to identify the factors for determining the decision to be insured or not and also estimated propensity score. The probit model results discuss that education, access to credit, chronic disease, insurance premium, awareness, distance to health service, and health service waiting time are significant determinates for CBHI insured, and also the estimated propensity score for full sample varies from 0.005 to 0.804 with a mean value of about 0.41. The PSM method was used to estimate the impact of CBHI on household welfare. Once controlling and balancing the dissimilarities in baseline covariates of sampled households, the PSM revealed that the insured households outpatient and inpatient service utilization (visit) increased by 2.6 times, reduced per-capita health expenditure by 17–14% points, increases the per-capita consumption of non-food items by 12–14% points, and increases the per-capita consumption of food items by 12–13% points in a given matching algorism compared to the counterparts.

Therefore, CBHI has enhanced service utilization, reduced the per-capita health expenditure, and increased consumption per capita, i.e., in general, it improved household welfare. In line with this, this study recommended that the government (like ministry of health) and concerned bodies (like NGOs) should give more emphasis, extend the coverage and accessibility of CBHI schemes, and promote the society to be insured in this scheme, by doing so, create awareness, provide the insurance at least premium, offered credit, strengthening education, established health facilities nearby, provide service efficiently.

## Data Availability Statement

The raw data supporting the finding of this article will be made available by corresponding author upon request, without restriction.

## Ethics Statement

Ethical clearance was obtained from the College of Business and Economics, Samara University. Confidentiality of the information was secured by excluding respondents' identifiers, such as names, from the data collection format. Finally, verbal informed consent was obtained from those who were in Chilga district and willing to participate in the study. Moreover, the results were recommended to be disseminated by the responsible bodies who were involved in health sectors.

## Author Contributions

SA contributed to conceptualization and formal analysis. DA contributed to methodology, funding acquisition, investigation, software, supervision, and writing the original draft. SS contributed to validation and data curtain. AB contributed to visualization, writing, reviewing, and editing the manuscript. All authors contributed to the article and approved the submitted version.

## Conflict of Interest

The authors declare that the research was conducted in the absence of any commercial or financial relationships that could be construed as a potential conflict of interest.

## Publisher's Note

All claims expressed in this article are solely those of the authors and do not necessarily represent those of their affiliated organizations, or those of the publisher, the editors and the reviewers. Any product that may be evaluated in this article, or claim that may be made by its manufacturer, is not guaranteed or endorsed by the publisher.

## Footnotes

^1^It is the lowest political administrative unit in the Ethiopia.
